# cblaster: a remote search tool for rapid identification and visualization of homologous gene clusters

**DOI:** 10.1093/bioadv/vbab016

**Published:** 2021-08-05

**Authors:** Cameron L M Gilchrist, Thomas J Booth, Bram van Wersch, Liana van Grieken, Marnix H Medema, Yit-Heng Chooi

**Affiliations:** 1 School of Molecular Sciences, The University of Western Australia, Crawley, WA 6009, Australia; 2 Bioinformatics Group, Wageningen University, Wageningen 6708PB, The Netherlands

## Abstract

**Motivation:**

Genes involved in coordinated biological pathways, including metabolism, drug resistance and virulence, are often collocalized as gene clusters. Identifying homologous gene clusters aids in the study of their function and evolution, however, existing tools are limited to searching local sequence databases. Tools for remotely searching public databases are necessary to keep pace with the rapid growth of online genomic data.

**Results:**

Here, we present cblaster, a Python-based tool to rapidly detect collocated genes in local and remote databases. cblaster is easy to use, offering both a command line and a user-friendly graphical user interface. It generates outputs that enable intuitive visualizations of large datasets and can be readily incorporated into larger bioinformatic pipelines. cblaster is a significant update to the comparative genomics toolbox.

**Availability and implementation:**

cblaster source code and documentation is freely available from GitHub under the MIT license (github.com/gamcil/cblaster).

**Supplementary information:**

Supplementary data are available at *Bioinformatics Advances* online.

## 1 Introduction

Complex biological processes are coordinated through the action of multiple distinct, yet functionally associated genes, which are often found physically collocated within genomic neighbourhoods as gene clusters. Gene clusters have been extensively studied in microbes for their ability to encode traits such as the biosynthesis of secondary metabolites, virulence, drug resistance and xenobiotic degradation; however, gene clustering is a phenomenon observed across all kingdoms of life (Chevrette *et al.*, 2020; Foflonker and Blaby-Haas, 2021; Nützmann *et al.*, 2018; Wang *et al.*, 2019). Given the relationship between genetic collocation and co-functionality (Lee and Sonnhammer, 2003; Michalak, 2008), it follows that we can detect gene clusters by directly searching for conserved instances of collocation across taxa. Indeed, tools such as MultiGeneBlast (Medema *et al.*, 2013) and clusterTools (Lorenzo de los Santos and Challis, 2017) have been developed precisely for this purpose. These tools take specific queries [nucleotide/protein sequences, hidden Markov model (HMM) profiles] and search them against genomic datasets, identifying any instances of collocated query hits. However, their utility is limited by the requirement to build and maintain local databases. In many cases, this is redundant given that most biological sequence data are deposited in publicly accessible online databases like the National Center for Bioinformatics Information (NCBI). It is also increasingly demanding as the amount of available data has grown rapidly in the past decade. Between January 2010 and September 2020, the total number of RefSeq genomes grew from 10 171 to 104 969 (NCBI RefSeq Growth Statistics page). This has two additional consequences: (i) maintaining an up-to-date local database has become significantly more difficult; and (ii) the size of search outputs has increased significantly. Therefore, there is a need for search tools that can leverage remote databases and visualize search outputs in an intuitive manner.

Here, we present cblaster, a Python-based tool to rapidly search for collocated protein-coding regions remotely by leveraging NCBI APIs, or locally within user-generated databases. cblaster is easy to use with both command line and graphical user interfaces (GUI). It generates fully interactive visualizations implemented in JavaScript and HTML that allow the user to intuit patterns from complex datasets, as well as textual outputs that can easily be incorporated into bioinformatic pipelines. cblaster is a significant update to the comparative genomic toolbox and is a launch pad for further functional and evolutionary analyses.

## 2 Fully remote searches against NCBI sequence databases

**Fig. 1.**
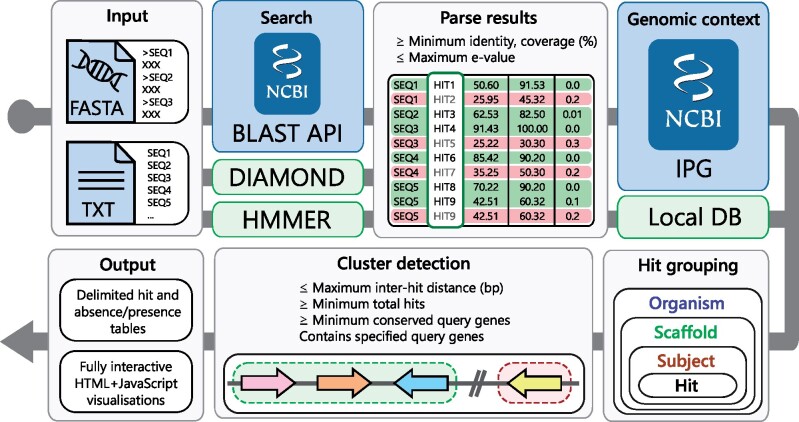
The cblaster search workflow. Input sequences are given either as a FASTA file or as a text file containing NCBI sequence accessions. They are then searched against the NCBI’s BLAST API or a local DIAMOND database, in remote (blue background) and local (green background) modes, respectively. BLAST hits are filtered according to user-defined quality thresholds. Genomic coordinates for each hit are retrieved from the IPG resource. Hits are grouped by their corresponding organism, scaffold and subjects. Finally, hit clusters are detected in each scaffold and results are summarized in output tables and visualizations

**Fig. 2.**
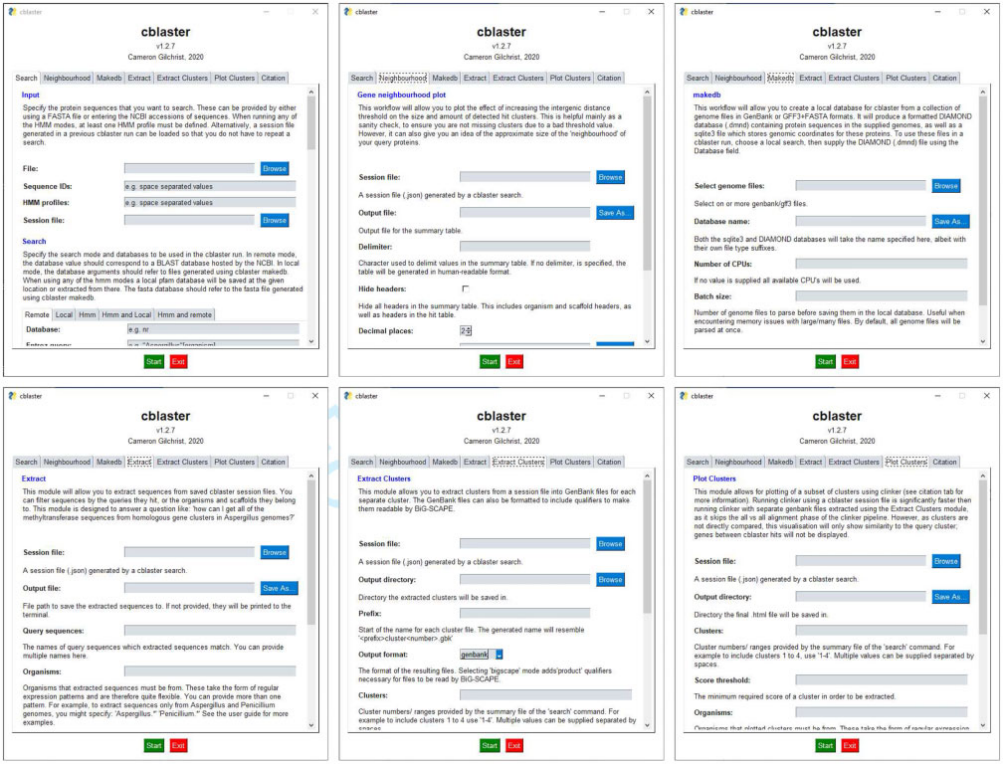
The cblaster GUI. Each panel represents a single cblaster module: cblaster search (top left) for performing searches against remote and local databases; cblaster gne (top right) for performing genomic neighbourhood estimation; cblaster makedb (bottom left) for building local databases from GenBank files and; cblaster extract (bottom right) for extracting FASTA files of specific groups of homologues

The cblaster search workflow is detailed in Figure 1 and can be launched either through the command-line interface (Table 1) or the GUI (Fig. 2). A search begins with the user providing either protein sequences (FASTA, GenBank or EMBL format) or a collection of valid NCBI protein sequence identifiers (i.e. accessions, GI numbers), that they believe may form a conserved gene cluster. If the latter is provided, sequences corresponding to each identifier are downloaded using the NCBI Entrez API (NCBI Resource Coordinators, 2017) prior to starting the search. Input sequences would typically be taken from the output of a cluster discovery pipeline such as antiSMASH (Blin *et al.*, 2019), or a resource such as the Minimum Information about a Biosynthetic Gene cluster database (MIBiG; Kautsar *et al.*, 2019). Sequences are uploaded to the NCBI BLAST API to launch a new search using BLASTp from the NCBI BLAST+ suite (Camacho *et al.*, 2009; NCBI Resource Coordinators, 2017). Optionally, an Entrez search query can be provided to pre-filter the search database, for example, to specify a taxonomic group of interest, resulting in vastly reduced search times. Every BLAST search is assigned a unique request identifier (RID) which remains active for 36 h. A cblaster search can be resumed at any point given a valid RID and the corresponding query sequences, such that search results can be retrieved at a later time if desired. This also allows cblaster to analyse searches launched through the BLAST website, which can be convenient when expecting long search times due to, for example, many query sequences or unfiltered search databases. cblaster can also save search sessions, which can be freely loaded back into the programme. The session file contains all data generated by cblaster during a search and can be used to re-detect clusters under new parameters or generate new visualizations without having to completely repeat entire searches.

**Table 1.** Overview of modules provided by the cblaster command-line interface

Module	Description
search	The main cblaster search workflow
makedb	Create local search databases from personal
	sequence data
gne	Gene neighbourhood estimation of cblaster
	search results
extract	Extract sequences from cblaster search results
gui	Launcher for the cblaster GUI
extract_clusters	Extract detected gene clusters from cblaster
	sessions
plot_clusters	Plot detected gene clusters using clinker
	(Gilchrist and Chooi, 2021)

The BLAST API is repeatedly polled until the search has completed, at which point search results are downloaded and filtered based on user-defined hit quality thresholds (minimum identity, coverage and maximum *e*-value). These thresholds are especially useful for narrowing down searches when expecting large result datasets. To retrieve the genomic context of remote BLAST hits, cblaster leverages the Identical Protein Groups (IPG) resource via the Entrez API (NCBI Resource Coordinators, 2017). The IPG database stores links from proteins to their exact genomic coordinates, meaning BLAST hits can be traced back to their genomic origins. To ensure no potential clusters are missed, for example, due to annotation error or fragmented genome assembly, all rows in the IPG table are saved and linked back to their original hits. Hits are grouped by their subject proteins, which are in turn grouped by genomic scaffold and organism. Finally, subjects are grouped into clusters if they contain hits to any required query sequences specified by the user (if any) and satisfy thresholds for maximum intergenic distance, minimum size and minimum hits per unique query sequence. After a gene cluster is finalized, it is assigned a unique numeric ID which is reported in the search output, which can be referenced in other cblaster modules.

Clusters are ranked using the scoring formula previously implemented in MultiGeneBlast (Medema *et al.*, 2013). Briefly, cluster similarity is calculated by

(1) S=h+i·s,

where *h* is the number of query sequences with BLAST hits, *s* is the number of contiguous gene pairs with conserved synteny and *i* is a weighting factor (default value 0.5) determining the weight of synteny in the similarity score.

## 3 Local searches against custom sequence databases

cblaster can also search local sequence databases. The local search workflow mirrors that of remote searches, with two key differences: (i) the DIAMOND search tool (Buchfink *et al.*, 2021) is used instead of BLASTp; and (ii) a local genome database is used in place of the IPG database to facilitate retrieval of the genomic coordinates of hit proteins.

A local search thus requires two databases to be created by the user: a formatted DIAMOND search database and a local genome database. cblaster provides a module, makedb, which can rapidly generate both databases from a collection of genome sequences simultaneously in a single command. Briefly, genome files are parsed using the BioPython (Cock *et al.*, 2009) or gffutils (https://github.com/daler/gffutils) libraries. cblaster then builds an SQLite3 database containing coordinates of genes within each genome, which can be loaded during local searches. Protein translations are extracted to a FASTA file from which the DIAMOND database is built; sequence headers correspond to unique database indices, allowing cblaster to retrieve information about sequences hit in a search.

## 4 Searching for functional composition using domain profiles

Although it is useful to identify clusters through sequence similarity, in some cases, this can be too restrictive. Another approach is to search for profile hidden Markov models (pHMMs), which correspond to functional domains. In this way, one can search for gene clusters containing hypothetical functionality as opposed to sequence similarity. This is particularly useful when using a ‘retro-biosynthesis’ approach, where biosynthetic gene clusters (BGCs) are identified based on functions informed by chemical structures (Cacho *et al.*, 2015). It can also be useful in cases where mutual sequence similarity between members of a protein family is low. For example, microbial terpene synthases typically show only weak sequence similarity, but can be readily identified through profile HMM searches (Komatsu *et al.*, 2008).

Similar functionality has been implemented previously in ClusterTools (Lorenzo de los Santos and Challis, 2017), where users can search local sequence databases for combinations of profile HMMs and protein sequences. cblaster provides a search mode, hmm, where users can perform searches using profile HMMs from the Pfam database (Mistry *et al.*, 2021) as queries instead of protein sequences. To do this, cblaster wraps the HMMER software package (Mistry *et al.*, 2013). In this workflow, query domain profiles are first extracted from the Pfam HMM profile database using hmmfetch and then searched against a local sequence database (FASTA file of amino acid sequences generated using the makedb module) using hmmsearch. This requires a local copy of the Pfam database; cblaster will automatically download the latest Pfam release if no copy is found. Due to the dependency on the HMMER package, this functionality is currently only supported on Linux and Mac systems. cblaster can also perform hybrid searches, where profile HMMs are searched alongside protein sequences.

## 5 Estimation of genomic neighbourhood size

**Fig. 3.**
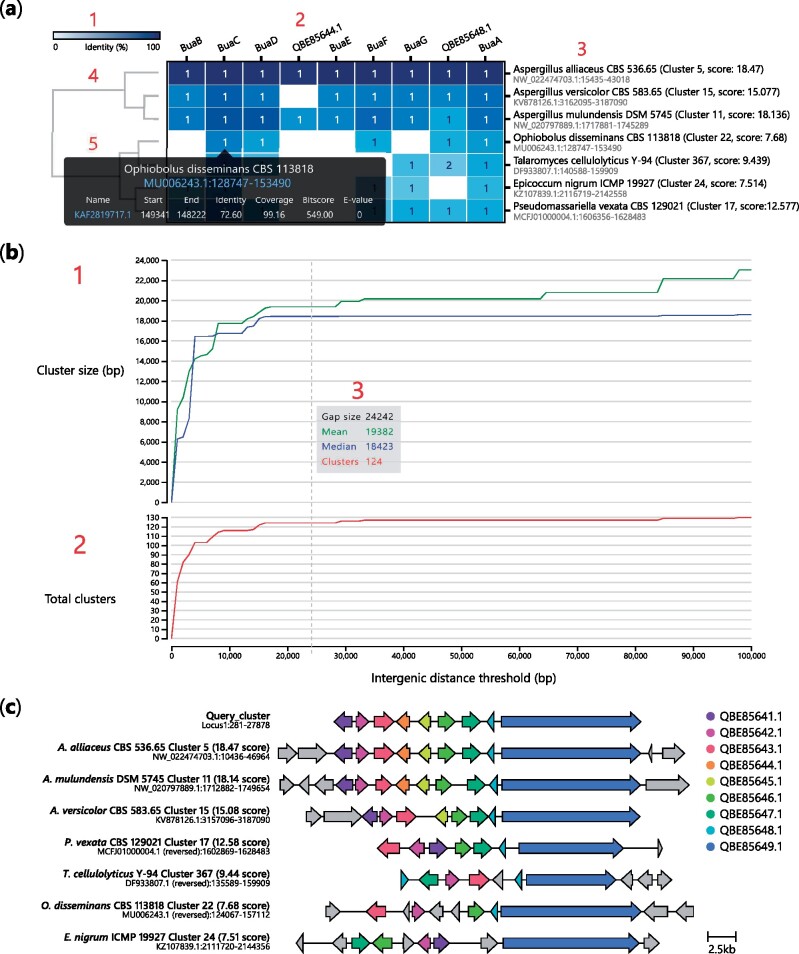
Interactive visualizations generated by cblaster. (**a**) Cluster heatmap visualization of cblaster search results: (1) heatmap colour bar indicating 0% (white) to 100% (blue) identity; (2) names of query sequences; (3) names of organism and scaffold locations of hit clusters; (4) dendrogram of hit clusters generated from their identity to query sequences; and (5) cell hover tooltip with detailed hit information including hyperlinks to genomic position on NCBI. (**b**) Gene neighbourhood estimation (GNE) visualization: (1) Plot of mean and median hit cluster sizes (bp) at different gap sizes; (2) Plot of total clusters at different gap sizes; (3) Hover tooltip showing values of mean and median cluster size (bp) and total clusters at a given gap size. (**c**) Visualization of gene clusters, inclusive of intermediate genes (grey colour), identified in (a) generated using the clinker tool via the plot_clusters module in cblaster

Gene cluster structure can vary greatly between organisms. For example, fungal and bacterial BGCs tend to be tightly packed, though in plants and some exceptions in fungi, genes encoding biosynthetic pathways are loosely clustered (Kessler *et al.*, 2020; Liu *et al.*, 2020b). In other cases, genes may be split across multiple loci as mini sub-clusters (Bradshaw *et al.*, 2013), or intertwined with clusters encoding other pathways to form superclusters (Wiemann *et al.*, 2013). During cluster detection, cblaster uses a user-defined threshold to determine the maximum distance between any two BLAST hits in a cluster. By default, a cluster is finalized if no new hit is found within 20 kbp of the previous hit. However, this value is arbitrary and may be inappropriate for some datasets, and so it is advisable to test the effect of changing this parameter on each dataset being analysed. cblaster provides the gne module, which leverages its ability to rapidly reload and recompute search sessions to robustly automate this analysis. When provided with a session file, gne iteratively performs cluster detection over a user-determined range of intergenic distance threshold values, then plots the total number of predicted clusters, as well as the mean and median cluster size (bp), at each value (Fig. 3b; Supplementary Additional File S2). These plots typically resemble logarithmic growth, steeply rising at low values but gradually levelling off at higher values. This makes it easy to determine sensible cut-offs that avoid liminal regions where small variability would have a large effect on the output.

## 6 Comprehensive output and fully interactive visualizations

cblaster offers rich summaries and visualizations of search results. cblaster’s outputs are available in both human-readable and character-delimited formats and can be easily incorporated into higher-level bioinformatic pipelines. This includes a summary of all detected clusters and a presence/absence table (here termed binary table), which shows the total number of hits in detected clusters per query sequence. By default, clusters are grouped by the organisms and scaffolds they are in; they can optionally be printed in rank order, with the most similar clusters appearing first.

Detected gene clusters can be extracted into GenBank format files directly from cblaster search sessions using the extract_clusters module. Users can filter clusters by their numeric IDs, the organism and scaffold they appear on, or by a minimum cluster similarity score. Optionally, features within each GenBank file can be formatted for interoperability with the biosynthetic gene similarity clustering and prospecting engine (BiG-SCAPE; Navarro-Muñoz *et al.*, 2020). In a similar vein, sequences within detected clusters can be extracted using the extract module. Sequences can be filtered by the query sequence/s they were hit by during the BLAST search, their organism or scaffold. The extract module can produce delimited summary tables as well as FASTA format files of all sequences matching the specified filters.

Search results are visualized as a cluster heatmap (Fig. 3a; Supplementary Additional File S1). Clusters are hierarchically clustered based on best hit identity values using the SciPy library (Virtanen *et al.*, 2020). Cells in the heatmap are shaded based on identity. The text inside each cell indicates if query sequences have multiple hits within a cluster. Figures can be freely panned and zoomed; columns (query sequences) and rows (clusters) can be hidden by clicking on their respective labels. Additionally, mousing over a cell in the cluster heatmap generates a tooltip that displays a summary of hits in the cluster for the corresponding query sequence. The tooltip also links to the exact cluster location on the NCBI’s graphic genome viewer.

Similarly, cblaster gne results are visualized as line charts (Fig. 3b) which can be panned and zoomed, with a tooltip showing the number of detected clusters, as well as the mean and median cluster size (bp), at different intergenic distance values.

cblaster visualizations are implemented as HTML documents that can be opened in the web browser. These documents contain scalar vector graphics images generated using the D3 visualization library (Bostock *et al.*, 2011), which can be exported for modification in vector image manipulation software. Any changes made within the visualization are reflected in the exported image. Additionally, cblaster can produce fully portable HTML documents can be generated, enabling results to be shared between different computers.

Finally, cblaster provides a module for analysing subsets of search results using the clinker tool (Gilchrist and Chooi, 2021), allowing visual comparison of the structure (inclusive of intermediate genes) of specific clusters of interest. This module mirrors the functionality of the extract_clusters module, whereby clusters can be filtered by ID, organism, scaffold and score.

## 7 Case studies

### 7.1 Case study 1: Analysing evolutionary relationships of chromopyrrolic acid-derived natural products

**Fig. 4.**
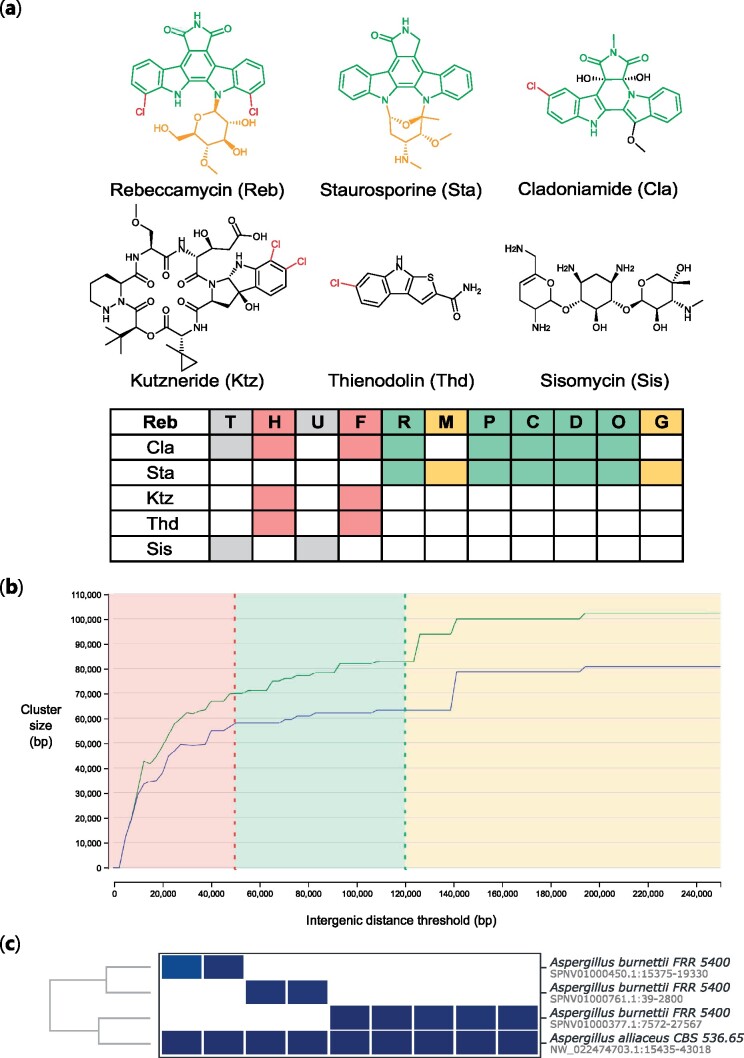
Application of cblaster to case studies in bacteria, plants and fungi. (**a**) A representative subset of the cblaster output in Case Study 1, highlighting the evolutionary relationships between rebeccamycin biosynthetic proteins (Reb) and other natural products. Structural features are highlighted according to conserved biosynthetic proteins (halogenases RebH and RebF in red, proteins involved in chromopyrrolic acid biosynthesis, RebR, RebP, RebC, RebD and RebO in green, glycosyltransferase, RebG, and methyltransferase, RebM, in yellow). The transporters RebT and RebU are shown in grey. (**b**) Genome neighbourhood estimation of plant triterpene BGCs. ‘Liminal’ region of plot shown in red, ‘stable’ region in green and upper limits in yellow. The accompanying total clusters plot (as in Figure 3b) is omitted from this figure, but follows the same pattern. (**c**) Using cblaster to piece together the burnettramic acids BGC in a fragmented *A.burnettii* genome

To assess cblaster’s ability to visualize evolutionary relationships, we searched for homologues of the *reb* BGC against a local database of characterized BGCs. The *reb* BGC is responsible for the production of the indolocarbazole rebeccamycin in strains of the actinobacterium *Lechevalieria aerocolonigenes* (Sánchez *et al.*, 2002). Indolocarbazoles are biosynthesized through the successive modification of chromopyrrolic acid, a dimer of tryptophan. Furthermore, they are closely related to other groups of tryptophan derived natural products, most significantly the indolotryptolines. As such, they represent a good case study to demonstrate the ability of cblaster to identify and group related families of BGCs. Using the cblaster makedb module, a local database was generated from GenBank sequences downloaded from the October 2019 release of the MIBiG database (Kautsar *et al.*, 2019). The database was queried using protein sequences extracted from the published rebbeccamycin BGC (GenBank accessionAJ414559) (Sánchez *et al.*, 2002).

cblaster identified 30 clusters from the MIBiG database, including all known indolotryptolines and indolocarbazoles, and the resulting dendrogram accurately reflected the relationship between the groups (Fig. 4a, Supplementary Figure S1, Case Study 1 in Supplementary Data). Interestingly, cblaster identified two additional groups containing *reb* homologues. Firstly, a group of BGCs encoding structurally unrelated compounds sharing tryptophan halogenases (RebH) and flavin reductases (RebF), including kutzneride (Fujimori *et al.*, 2007), thienodolin (Wang *et al.*, 2016) and ulleungmycin (Son *et al.*, 2017). Although structurally diverse, all members of this group contain a halogenated tryptophan motif in the final structure. Secondly, a group sharing homologues of one or both or the *reb* transport proteins (RebT and RebU), including the glycosides gentamicin (Huang *et al.*, 2015; Unwin *et al.*, 2004) and sisomicin (Hong *et al.*, 2009).

Although these are only surfacelevel observations, the results demonstrate the ability of cblaster to rapidly highlight both apparent and less conspicuous evolutionary relationships.

### 7.2 Case study 2: Genomic neighbourhoods and the biosynthesis of triterpenes in plants

Recent studies have highlighted the importance of genomic neighbourhoods in the evolution of plant development and metabolism (Mihelčić *et al.*, 2019; Nützmann *et al.*, 2016). Brassicacaea for example, have evolved to biosynthesize a range of triterpene products through the process of gene shuffling in plastic regions of the genome (Liu *et al.*, 2020a). During triterpene biosynthesis, 2,3-oxidosqualine is cyclized by an oxidosqualine cyclase (OSC). The cyclized product is then decorated through oxidation by cytochrome P450s or acetylation by acetyltransferases. The differing combinations of cyclases and tailoring enzymes drive the remarkable diversity of terpene products.

In most studies, the definition of genomic neighbourhoods is arbitrary, i.e. set to a reasonable, but static value (e.g. selecting five genes either side of the gene of interest). This is analogous to antiSMASH (Blin *et al.*, 2019) prediction, where BGC boundaries are set a fixed distance up- and downstream of the core genes. We decided to take advantage of cblaster’s ability to rapidly recompute outputs to define genomic neighbourhoods in a more robust fashion.

To identify triterpine BGCs, we took the sequences of proteins involved in thalianol biosynthesis in *Arabadopsis thaliana* and searched them against the NCBI database. As described in Field *et al.* (2011), this included a thalianol synthase (At5g48010), two cytochrome P450s (At5g48000 and At5g47990) and an acyltransferase (At5g47980). We then used the gne module to iteratively predict BGCs based on increasing intergenic distance values. From these data, we can readily identify suitable values for the intergenic distance thresholds. Intergenic distances below 50 kb are unsuitable, as they are within the ‘liminal’ region of the curve. Notably, the default threshold of 20 kb lies well within this region, indicating that many BGCs would be missed by cblaster if this parameter were not changed. Values above 475 kb lead to a massive spike in the mean cluster size while cluster count remains stable, indicating that homologues are being hit from more distant regions of the genome. Between these two values, there are a number of stable values suitable for analysis, from a conservative threshold of 50 kb to a more liberal threshold of 120 kb (Fig. 4b). As such, gne allows the user to rapidly assess suitable intergenic distance values that avoid liminal regions where small variability would have a large effect on the output.

### 7.3 Case study 3: Identification of BGCs in fungi

Previously, we reported the *bua* BGC, responsible for the production of the burnettramic acids in *Aspergillus burnettii* (Li *et al.*, 2019). Due to a fragmented genome assembly, initially we could only identify a short fragment of *bua* containing the genes *buaA* and *buaE*, encoding a hybrid polyketide synthase-nonribosomal peptide synthetase (PKS-NRPS) and proline hydroxylase, respectively. As BGCs are often conserved across multiple species, comparative genomics can be used in this scenario to identify the full set of biosynthetic genes (Cacho *et al.*, 2015). Thus, we employed cblaster to search for co-located *buaA/E* sequence homologs across publically available genomes in the NCBI database. This resulted in the identification of complete homologous BGCs in other *Aspergillus* spp., including *Aspergillus alliaceus*, which allowed us to map back to other truncated genomic scaffolds in the *A.burnettii genome* and reconstruct the full *bua* BGC (Fig. 4c), which we then experimentally characterized.

In another study, we used cblaster to identify BGCs encoding the biosynthesis of natural products with 1-benzazepine scaffolds in *Aspergillus* spp. using genes from the nanangelenin biosynthetic pathway (Li *et al.*, 2020). Searching for collocated homologues of the bimodular NRPS, NanA, and the indoleamine-2,3-dioxygenase, NanC, revealed related BGCs in the genomes of six *Aspergillus* species. In addition, the different clusters encoded different complements of tailoring enzymes, such as methyltransferases and P450s, indicating that these BGCs are likely responsible for the production of novel analogues of the nanangelenin family.

More recently, we reported *hkm*, the BGC encoding the biosynthesis of the hancockiamides, a family of phenylpropanoid piperazines isolated from *Aspergillus hancockii* (Li *et al.*, 2021). While the BGC itself was identified through manual BLASTp searches of the *A.hancockii* genome, using cblaster enabled us to identify homologous gene clusters in several other fungal species.

### 7.4 Case study 4: Identifying drimane sesquiterpenoid BGCs through profile searches

We recently investigated a family of drimane sesquiterpenoids, the nanangenines, isolated from *Aspergillus nanangensis* from section *Jani* (Lacey *et al.*, 2019). Previous work by Shinohara *et al.* (2016) identified a drimane synthase, AstC, involved in the biosynthesis of the astellolides, a family of drimane sesquiterpenoids. AstC is a novel member of the terpene synthase family and shows similarly to haloacid dehydrogenase (HAD)-like hydrolases. The presence of acyl side chains in the nanangenines led us to hypothesize that their biosynthesis would require a drimane synthase, similar to AstC, and either a fatty-acid synthase or PKS. In the study, we performed BLAST searches to identify AstC homologues in the *A.nanangensis* genome and manually filtered the results for only those found collocated with a PKS or FAS. This led to the identification of the candidate cluster (GenBank accessionMT024570.1) that we presented in the paper, containing a PKS (FE257_006541) and an AstC homologue (FE257_006542).

cblaster provides a search mode, hmm, which allows for searches against local sequence databases using functional domain profiles from the Pfam database (Mistry *et al.*, 2021). Our functional hypothesis for the biosynthesis of the nanangenines presented a perfect use case for this search mode. To demonstrate this, we attempted to directly identify the same candidate gene cluster in the genome using the domain profiles for a beta-ketoacyl synthase domain (Pfam accession PF00109) and a HAD-like hydrolase domain (Pfam accession PF13419). This search resulted in just two cluster hits, including our manually identified candidate cluster.

We then performed comparative genomics analysis to further support the validity of our candidate cluster. Though drimane sesquiterpenoids are produced by several Aspergilli, those with acyl side chains are unique to species in section *Usti*. We used cblaster to search for homologues of our candidate cluster, both remotely in the NCBI BLAST databases, as well as locally against section *Usti* species with genomes deposited in other sources. As a result, we found homologues spanning up to six genes in *Aspergillus ustus*, *Aspergillus calidoustus*, *Aspergillus insuetus* and *Aspergillus pseudodeflectus*, all of which are members of section *Usti* as well as known producers of acyl drimane sesquiterpenoids. Further analyses of these clusters using clinker (Gilchrist and Chooi, 2021) gave us confidence that our candidate BGC does indeed encode the biosynthesis of the nanangenines.

Though further studies are necessary to fully characterize this biosynthetic pathway, it shows how cblaster has quickly become an indispensable part of this research workflow.

### 7.5 Case study 5: Benchmarking comparison with MultiGeneBlast

The closest comparable tool to cblaster currently available is MultiGeneBlast (Medema *et al.*, 2013). MultiGeneBlast does not have remote search functionality. However, cblaster improves upon its local search functionality through its implementation in Python 3, the use of DIAMOND (Buchfink *et al.*, 2021) and SQLite3 databases. In order to demonstrate the performance difference between the two tools, we benchmarked their speed and accuracy by testing their ability to identify characterized BGCs in a database of *Aspergillus* genomes.

Initially, 88 BGCs were retrieved from the MIBIG database (Kautsar *et al.*, 2019); after removing duplicates and any records consisting only of single genes, 80 remained in the query collection (Supplementary Table S1). The search database was built using the whole-genome assemblies of 151 *Aspergillus* species, as well as 92 BGCs deposited directly as gene clusters in the NCBI’s nucleotide database. The retrieved genomic regions containing the clusters for ferrichrome in *Aspergillus oryzae* (BGC0000900) and *Aspergillus niger* (BGC0000901), and squalestatin S1 in *A.* sp. *Z5* (BGC0001839), lacked sequence feature annotations on the NCBI; in these cases, the annotated MIBiG entries were used instead. This resulted in a total of 243 sequence records containing 1 828 599 gene sequences. Using default settings, MultiGeneBlast took 2801.12 s (∼46.69 min) to construct a local database from these sequence records. In comparison, cblaster was able to construct its databases in just 121.42 s (∼2.02 min), a 27x speedup over MultiGeneBlast. This could be due to several reasons. cblaster uses the robust GenBank parser from the BioPython library (Cock *et al.*, 2009) to parse input sequences, whereas MultiGeneBlast uses a custom implementation. However, the largest difference in speed is most likely due to the utilization of parallel processing in cblaster which allows multiple genomes to be parsed simultaneously. Another consideration is that while cblaster uses an SQLite3 database to store genomic coordinates, MultiGeneBlast instead stores this information in the description lines of records in a FASTA file. As MultiGeneBlast parses genomes sequentially and appends directly to this FASTA file during database construction, only a single genome is kept in memory at any one time. In cblaster, however, parsed genomes are stored in memory until some user-defined threshold of genomes is reached, at which point all stored genomes are written to the database and memory is freed. While no limit was used in this test, batching of genomes may incur a slight performance penalty.

**Fig. 5.**
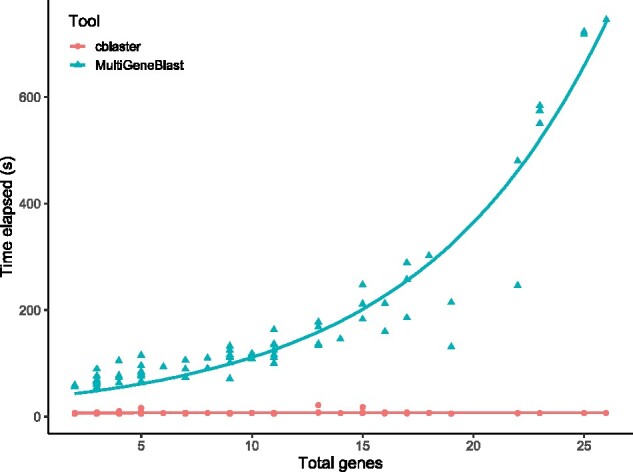
Time taken (s) to search query clusters from MIBiG containing differing numbers of genes against a database of *Aspergillus* genomes using MultiGeneBlast (blue circles) and cblaster (red triangles)

We then searched the constructed databases for each query BGC. In all cases, both cblaster and MultiGeneBlast successfully retrieved the query cluster from the search database. Search times in MultiGeneBlast grow exponentially worse as the number of genes within the query BGC increases, whereas searches in cblaster stay relatively constant (Fig. 5, raw data in Supplementary Table S1). MultiGeneBlast took a total of 13 130.21 s (∼3.65 h) to complete all searches, whereas cblaster completed all searches in just 584.01 s (∼9.73 min), a 22.48x speedup. The slowest MultiGeneBlast search involved the sterigmatocystin cluster from *Aspergillus ochraceoroseus* (BGC0000011, containing 26 genes), which took 745.33 s (∼12.42 min); in cblaster, the same search took only 6.96 s, a 107x speedup. Even the fastest MultiGeneBlast searches were considerably faster in cblaster; for example, searching for the biotin BGC from *Aspergillus nidulans* (BGC0001238) took 54.5 s in MultiGeneBlast, but just 5.32 s in cblaster. As the sequence search step is the most time-consuming step of the search process, the difference in performance is most likely due to the underlying search tools. cblaster uses DIAMOND (Buchfink *et al.*, 2021), which has been shown to be significantly faster than BLASTp (Camacho *et al.*, 2009), used in MultiGeneBlast. Moreover, MultiGeneBlast uses a bundled BLASTp binary, which is significantly slower than more recent releases of the BLAST+ suite (2.2.24 of the bundled binary versus 2.12.0 at time of writing). As in the database construction phase, another advantage of cblaster searches is the use of the SQLite3 database for retrieving the genomic coordinates of hit sequences. MultiGeneBlast stores positional information in a FASTA file, which must be read into memory and inefficiently queried for each individual sequence hit during a search, whereas in cblaster, all necessary information can be retrieved from a single SQL query.

cblaster is thus faster than MultiGeneBlast in both the construction and searching of local databases. It is worth noting, however, that the primary feature of cblaster is remote search, and as such it requires no local database to be created when searching public NCBI databases. While these benchmarks show that cblaster is a significant improvement upon MultiGeneBlast in local searches, they do not capture the time taken to construct and maintain local databases. Remote search times may vary depending on several factors, such as server load or query size. However, the convenience of running comprehensive searches directly against the entire NCBI database will outweigh this speed difference for many users.

All performance benchmarks were generated using the perf command-line utility on a workstation computer with an Intel(R) Xeon(R) E5-2630 CPU (2.40 GHz) and 64 gb of RAM. Further details about the benchmarking process, including custom Bash and Python scripts, have been made available on GitHub (github.com/gamcil/cblaster_benchmarks).

## 8 New approaches and practical applications

The case studies above demonstrate that cblaster is a powerful tool for identifying homologous gene clusters from the NCBI or local databases. It is possible to query known gene clusters to expand existing families, or user-specified combinations of genes that are predicted to function together. Currently, the most widely used tools for conducting gene cluster homology searches, MultiGeneBlast (Medema *et al.*, 2013) and clusterTools (Lorenzo de los Santos and Challis, 2017), depend on local sequence databases. cblaster allows remote searching of NCBI sequence databases, eliminating this requirement and reducing the computational burden (Table 2).

**Table 2.** Feature comparison of software tools for gene cluster homology searches

Feature	cblaster	MultiGeneBlast	clusterTools
Query input	Proteins, HMM profiles	Proteins, nucleotides	Proteins, HMM profiles
Searches against remote databases	X	—	—
Genomic neighbourhood estimation	X	—	—
Extraction of hit sequences	X	—	—
Interactive visualizations	X	X	—
Allows input of personal sequence data	X	X	X
Allows creation of local search databases	X	X	X
GUI available	X	X	X

cblaster provides outputs that can be easily adapted for downstream analyses. Delimited formats can be accessed as spreadsheets, making them accessible to all researchers regardless of bioinformatics experience. The binary table can be sorted and filtered post-analysis, which is particularly useful for narrowing large datasets to focus on individual genes or organisms of interest. In this way, data can be easily extracted to generate simple graphs (e.g. generic distribution, ubiquity of different homologues or average cluster size), build distance matrices and dendrograms. Results tables from multiple searches can easily be concatenated to combine results from local and remote searches. cblaster extract can be used to quickly obtain specific homologue sequences for downstream phylogenetic and structural analyses.

By using heatmaps as opposed to more complex gene diagrams, information about the diversity and conservation of encoded proteins is accessible at a glance (Fig. 3a). Grouping gene clusters by identity highlights blocks of homologous proteins, facilitating the designation of cluster boundaries and identification of core biosynthetic genes and functional sub-clusters. This is a useful proxy for functional and evolutionary relationships, as shown in Case Study 1 above. In this manner, cblaster can uncover deep-seated evolutionary relationships that may not be immediately apparent, such as proteins or sub-clusters that have been co-opted between different pathways. Furthermore, cblaster can generate comparative gene cluster visualizations using clinker (Gilchrist and Chooi, 2021). These visualizations show the detected clusters in their genomic context, enabling a fuller view of the overall conservation of cluster architecture across taxa.

cblaster gne can estimate the size of genomic neighbourhoods to help evaluate the robustness of cluster prediction (Fig. 3b). This enables the user to identify suitable intergenic distance values that limit missing data without inadvertently introducing false positives. In addition, recent work has highlighted the importance of genomic neighbourhoods in the evolution of gene clusters (Liu *et al.*, 2020a). The distribution of genomic neighbourhood sizes, as calculated by gne, provides insight into the conservation of gene cluster topology; a broader distribution indicates a more dynamic architecture. This aspect is likely to be of particular interest in the study of plants and other eukaryotes where gene clusters are more variable in gene density, displayed in Case Study 2 above.

In summary, cblaster can uncover homologous gene clusters and evolutionary relationships at a pace that far exceeds other available software and has become an indispensable tool in both our own research into the BGCs of fungi and bacteria (Lacey *et al.*, 2019; Li *et al.*, 2019, 2020, 2021; Morshed *et al.*, 2021) and in others (Jung *et al.*, 2021; Williams *et al.*, 2021). We foresee cblaster being used to uncover novel variants of known pathways, or to prospect for new combinations of biological parts for use in synthetic biology (Gilchrist *et al.*, 2018; Sun *et al.*, 2015; Wang *et al.*, 2013). cblaster’s versatility means it can be applied to any project that requires the study of two or more collocated genes and it is accessible to all researchers regardless of their experience with bioinformatics.

## Software and data availability

cblaster is implemented in Python, with visualizations written in JavaScript and HTML. Source code is freely distributed on GitHub (github.com/gamcil/cblaster) under the MIT license. A comprehensive usage guide as well as API documentation is available online (cblaster.readthedocs.io/en/latest).

## Funding

C.L.M.G. is supported by the Australian Government Research Training Program PhD scholarship. Y-H.C is supported by an Australian Research Council Future Fellowship (FT160100233). This work was funded in part by the Cooperative Research Centres Projects scheme (CRCPFIVE000119).

*Conflict of Interest*: MHM is a co-founder of Design Pharmaceuticals and a member of the scientific advisory board of Hexagon Bio.

## Supplementary Material

vbab016_Supplementary_Data
