# Supplementary material for: cblaster: a remote search tool for rapid identification and visualization of homologous gene clusters
Source: Bioinform Adv. 2021 Aug 5;1(1):vbab016. doi: 10.1093/bioadv/vbab016 (PMC9710679; doi:10.1093/bioadv/vbab016)
Supplement: vbab016_Supplementary_Data [file vbab016_supplementary_data.zip › CaseStudy2_Output.html]

cblaster


cblaster

This plot shows the effect of increasing the intergenic distance threshold
(--gap argument in cblaster) on both the total amount of predicted
clusters, as well as the mean and median cluster size (bp).

---

If you found cblaster useful, please cite:

```
						Gilchrist, C.L.M, 2020. cblaster: a Python toolkit for detecting co-located BLAST hits.
					
```

---

Zoom with middle mouse, pan by clicking and dragging.

Save SVG
