# Supplementary material for: cblaster: a remote search tool for rapid identification and visualization of homologous gene clusters
Source: Bioinform Adv. 2021 Aug 5;1(1):vbab016. doi: 10.1093/bioadv/vbab016 (PMC9710679; doi:10.1093/bioadv/vbab016)
Supplement: vbab016_Supplementary_Data [file vbab016_supplementary_data.zip › supplementary.pdf]

`cblaster`: a remote search tool for rapid identification and visualisation of homologous gene clusters Supplementary Information

Cameron L.M. Gilchrist[1*], Thomas J. Booth[1], Bram van Wersch[2], Liana van Grieken[2], Marnix H. Medema[2*], Yit-Heng Chooi[1*]

[1]School of Molecular Sciences, The University of Western Australia, 35 Stirling Hwy, Crawley, 6009

[2]Bioinformatics Group, Wageningen University, Droevendaalsesteeg 1, 6708PB Wageningen, The Netherlands

[*]Correspondence: cameron.gilchrist@research.uwa.edu.au, marnix.medema@wur.nl, yitheng.chooi@uwa.edu.au

List of Figures

1 Relationship between the rebeccamycin (*reb*) biosynthetic gene cluster (BGC) and other known BGCs 2

2 Genomic neighbourhood estimation plot 3

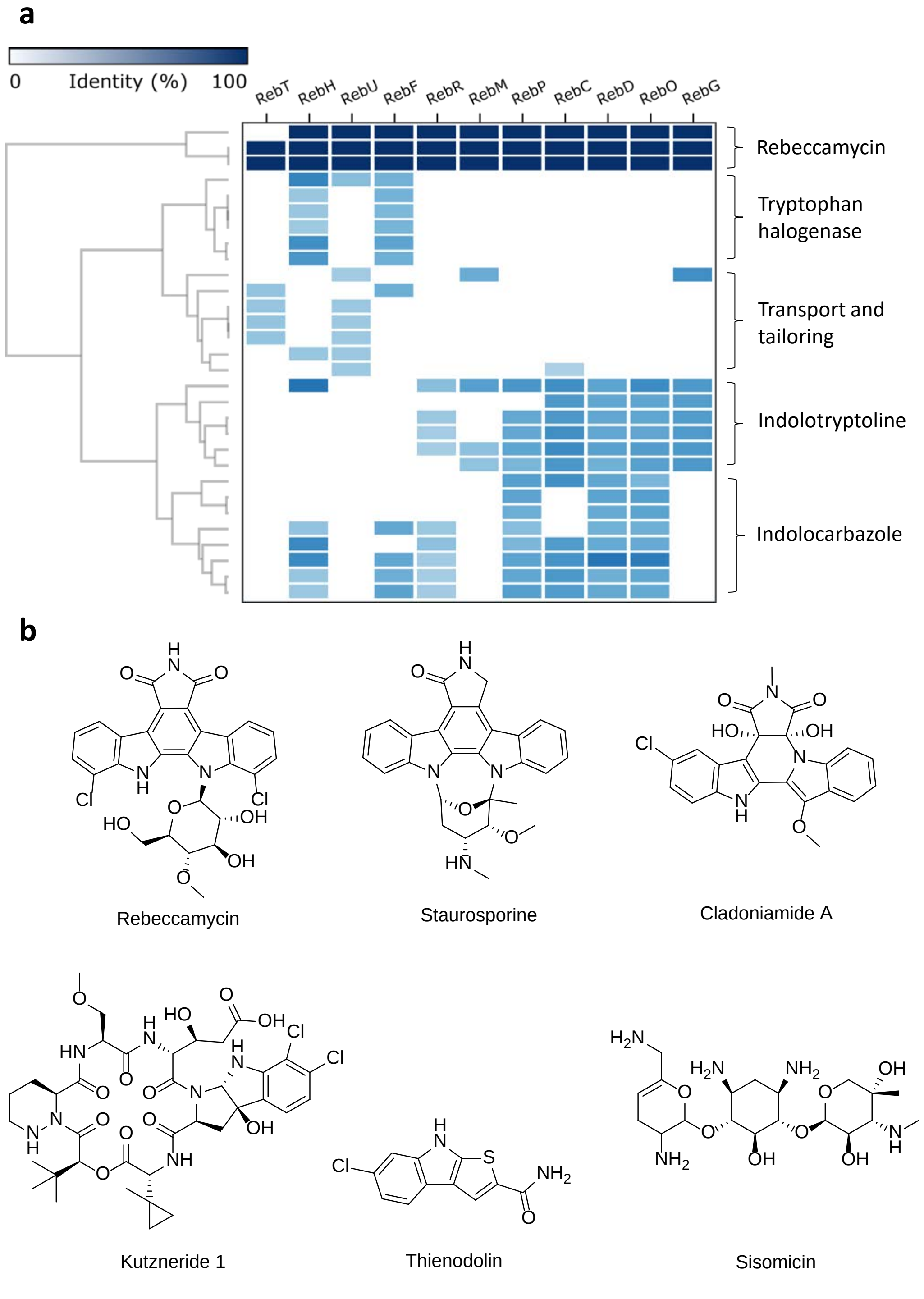

Figure 1: Relationship between the rebeccamycin (*reb*) biosynthetic gene cluster (BGC) and other known BGCs, showing a) the plot as produced by `cblaster` showing the homology of *reb* proteins to those of other BGCs and b) products of rebecamycin and related BGCs as predicted by `cblaster`.

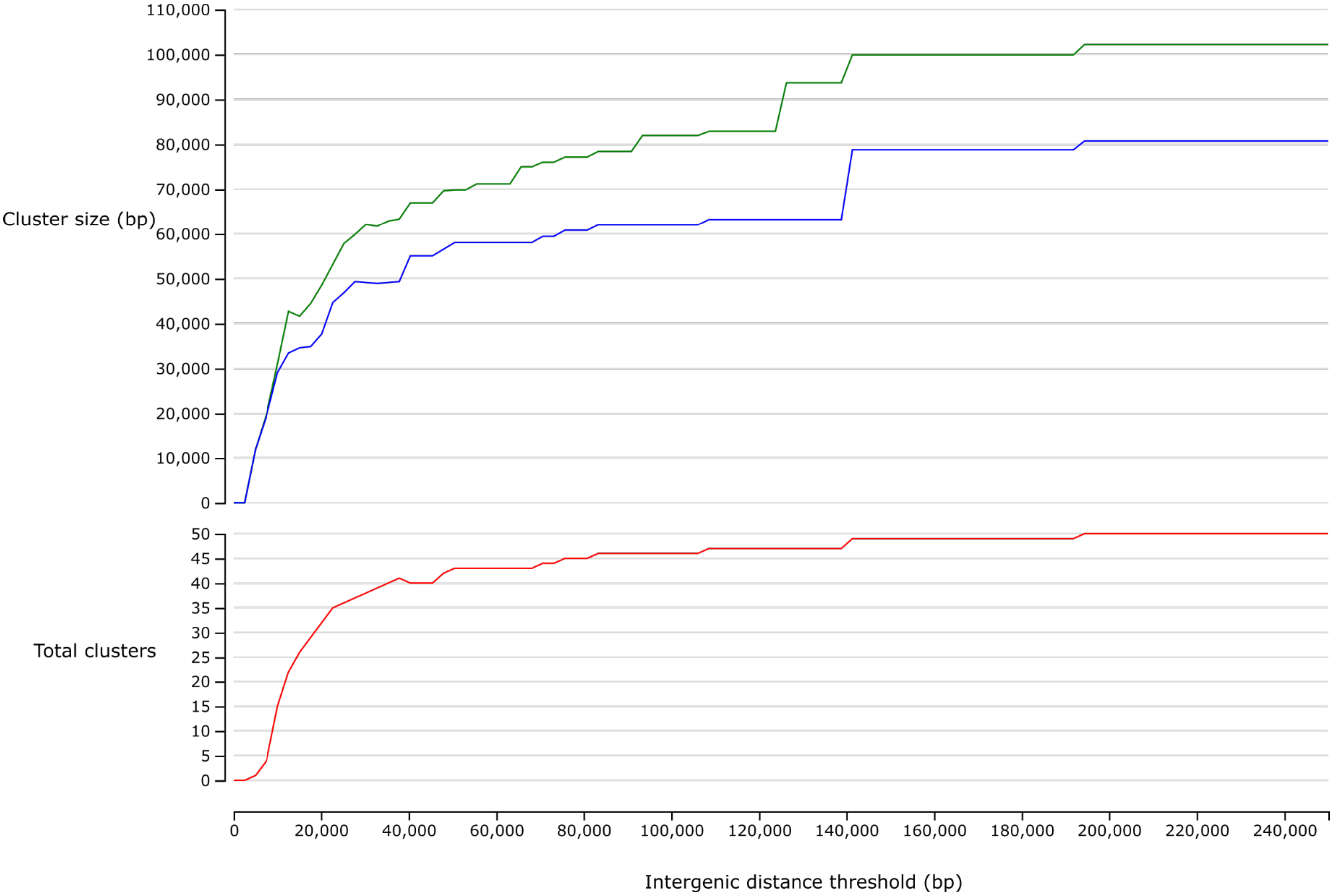

Figure 2: Genomic neighbourhood estimation as calculated by cblaster showing the total number of clusters (red) and the mean (green) and median (blue) cluster sizes for given intergenic distances.
